# Brain cavernous hemangioma mimicking radiation‐induced necrosis in a patient with non‐small cell lung cancer

**DOI:** 10.1111/1759-7714.13494

**Published:** 2020-05-29

**Authors:** Shinkichi Takamori, Takashi Seto, Mikako Jinnouchi, Taichi Matsubara, Naoki Haratake, Naoko Miura, Ryo Toyozawa, Masafumi Yamaguchi, Mitsuhiro Takenoyama

**Affiliations:** ^1^ Department of Thoracic Oncology National Hospital Organization Kyushu Cancer Center Fukuoka Japan; ^2^ Department of Radiology National Hospital Organization Kyushu Cancer Center Fukuoka Japan

**Keywords:** Cavernous hemangioma, non‐small cell lung cancer, radiation‐induced necrosis

## Abstract

In patients with non‐small cell lung cancer (NSCLC), stereotactic radiotherapy (SRT) is one of the standard therapies for those suffering with intracranial metastatic NSCLC. Radiation‐induced necrosis (RIN) sometimes occurs as the result of the delayed effects of SRT. The magnetic resonance imaging (MRI) of RIN typically shows hypointense and hyperintense lesions on T1‐ and T2‐weighted images, respectively. We herein report a patient with a growing brain cystic lesion mimicking RIN adjacent to a post‐radiation brain metastasis from NSCLC harboring anaplastic lymphoma kinase rearrangement. The patient underwent surgical resection of the brain tumor because of the symptoms. The pathological diagnosis was cavernous hemangioma, and the pathological findings were an encapsulated nodular mass composed of dilated, cavernous vascular spaces with no residual tumor or recurrence. Clinicians should be aware of the possibility for the development of a brain cavernous hemangioma following SRT in NSCLC patients.

## Introduction

Lung cancer is one of the most fatal malignancies worldwide, and non‐small cell lung cancer (NSCLC) accounts for approximately 85% of all lung cancer cases.^1^ In patients with NSCLC, brain metastases are diagnosed in approximately 40% cases during the course of the disease.^2^ Patients with brain metastases from NSCLC often develop in‐field and/or out‐of‐field progression. Nardone *et al*.^3^ reported that 9% of patients with brain metastases from NSCLC showed in‐field progression and 22% developed new out‐of‐field brain metastases. Stereotactic radiotherapy (SRT) is one of the standard treatments for patients suffering with a limited number of intracranial metastatic NSCLC,^4^ and radiation‐induced necrosis (RIN) sometimes occurs as the result of the delayed effects of SRT.^5^ Magnetic resonance imaging (MRI) is a sensitive screening test for brain RIN, typically indicating lesions which are hypointense on T1 and hyperintense on T2.

Cavernous hemangiomas are one of the vascular benign tumors composed of abnormally enlarged vascular structures, and it has been suggested that the growth is caused by the surrounding hemorrhagic cyst cavity and/or endothelial cell hyperplasia.^6^, ^7^ Cavernous hemangiomas are commonly observed in skin, liver, and soft tissues, and reported to be rare in the brain in adulthood.^8^, ^9^ Although there are several known causes for cavernous hemangioma, including radiation therapy and gene mutations or deletions, the pathogenesis of the disease is still not fully understood.^10^, ^11^, ^12^, ^13^ Cavernous hemangiomas are diagnosed by MRI scan which typically shows hypointense and hyperintense lesions on T1‐ and T2‐weighted images, respectively,^14^ similar to that of brain RIN. We herein report a NSCLC patient with a growing cavernous hemangioma in the brain mimicking RIN.

## Case report

**Figure 1:**
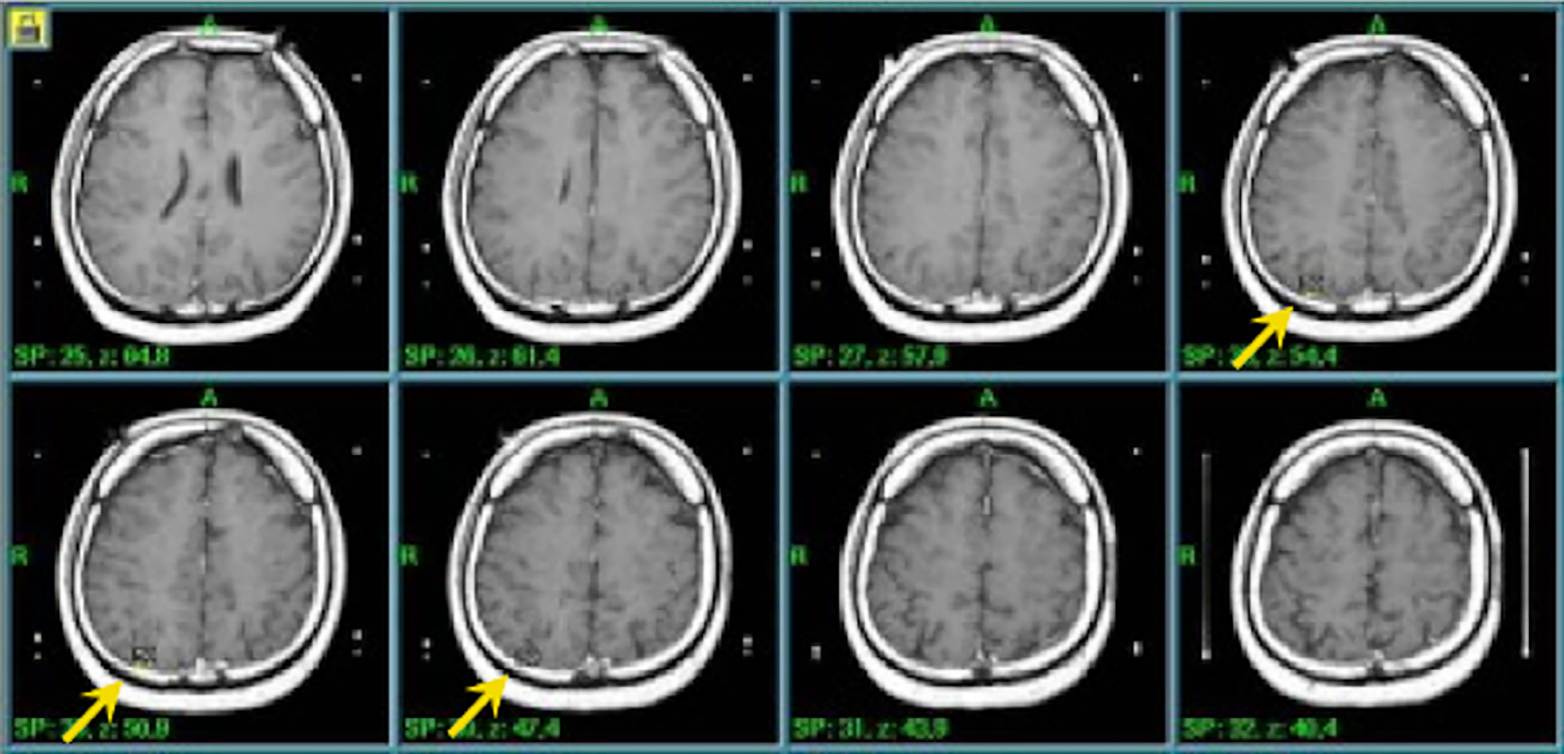
Planning computed tomography (CT) images of stereotactic radiotherapy with isodose distributions.

**Figure 2:**
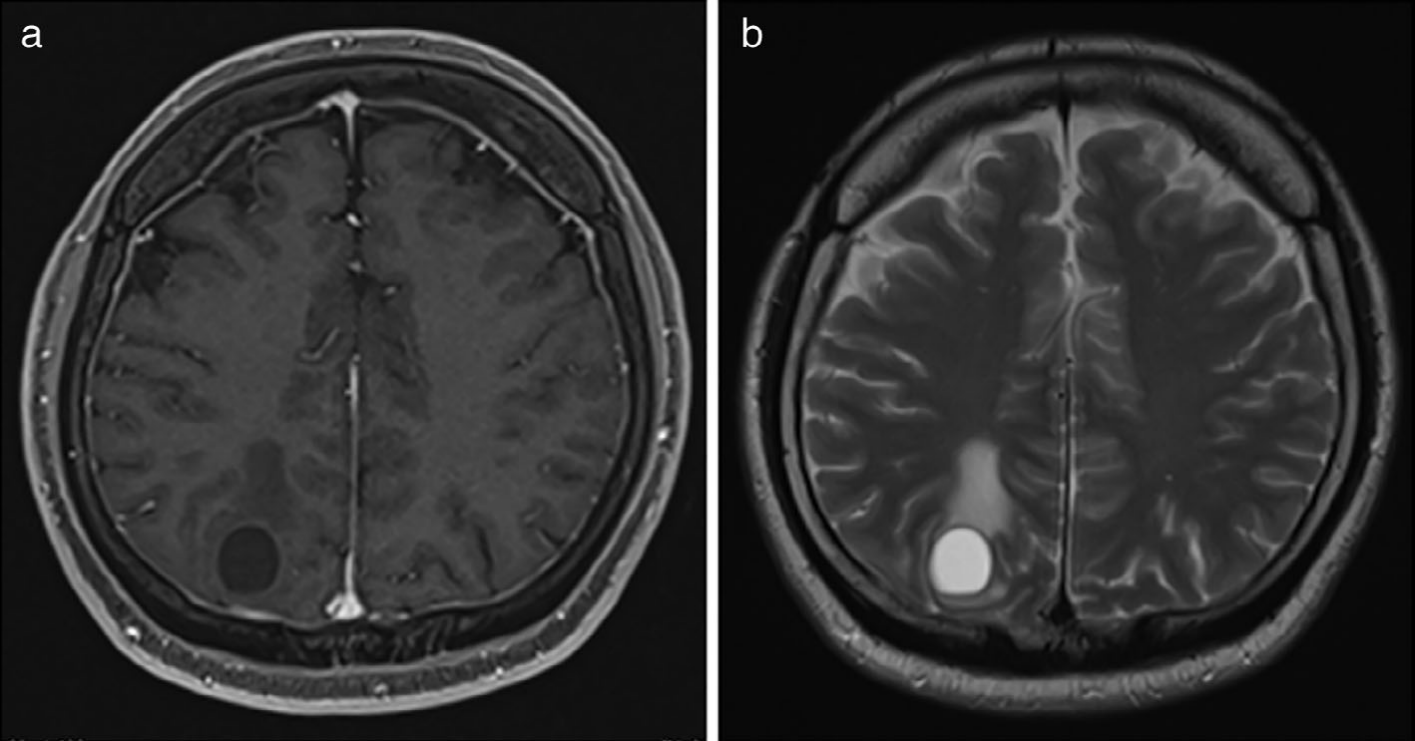
Magnetic resonance imaging (MRI) of brain cavernous hemangiomas mimicking radiation‐induced necrosis. The lesion was hypointense on (**a**) T1‐weighted image and hyperintense on (**b**) T2‐weighted image.

A 36‐year‐old man was diagnosed with primary lung adenocarcinoma in the right lower lobe (cT2aN1M0, cStage IIB). He underwent right middle and lower lobectomy in November 2011, and anaplastic lymphoma kinase (ALK) rearrangement was detected by fluorescence in situ hybridization. The patient participated in a clinical trial and alectinib 600 mg/day was administered from April 2011. In April 2018, brain MRI scan showed a brain metastasis in the right lobe of the brain. Stereotactic radiotherapy (SRT) (30 Gy/3 Fr) with prescribed 50% isodose line of maximum dose fitting to planning target volume (PTV) was performed for the single brain metastasis in May 2018 (Fig 1). The brain MRI scan showed that a cystic lesion had appeared adjacent to the post‐radiation brain metastasis, and the lesion was hypointense and hyperintense on T1‐ and T2‐weighted images, respectively (Fig 2). We consulted with a neurosurgeon, and the brain lesion was diagnosed as brain necrosis based on the radiological findings. After four months, the cystic lesion and the surrounding edema had increased in size. The patient had an epileptic seizure caused by optical stimulation. Therefore, we again consulted a neurosurgeon, and surgical resection was indicated. In January 2019, he underwent surgical resection of the growing brain lesion, and the pathological diagnosis was cavernous hemangioma. The specimen showed no residual tumor or recurrence. The patient had a favorable postoperative course, and has been attending to our hospital for one year.

## Discussion

Cavernous hemangiomas are one of the vascular benign tumors composed of abnormally enlarged vascular structures, and rare in the brain.^6^, ^7^, ^8^, ^9^ Approximately 86% of cavernous hemangiomas have been reported to be located supratentorially.^8^ With regard to the lesion growth of cavernous hemangioma, several articles have previously suggested that the growth is caused by the surrounding hemorrhagic cyst cavity and/or endothelial cell hyperplasia and/or the budding of capillaries.^6^, ^15^ Of note, the MRI findings are similar between cavernous hemangioma and RIN.^16^ Regarding the cause of cavernous hemangiomas, radiation treatment used for other medical conditions has been reported to be one of the causes of the disease.^10^ Gastelum *et al*.^10^ reported that the median latency time for detection of the brain cavernous hemangioma after cranial radiation therapy was 12 years, and that the cumulative incidence was 3% at 10 years post cranial radiation therapy and 14% at 15 years. In our case, the latency time was only 0.5 year, which is very rare. In patients with NSCLC harboring *ALK* rearrangement, it is also notable that cystic brain metastases have been reported.^17^ Given that cystic brain metastases usually show as hypointense lesions on T1‐weighted images and hyperintense on T2‐weighted images, it is difficult to diagnose cavernous hemangiomas as the clinical course of brain lesions have similarities with those of brain metastases from NSCLC.^18^, ^19^

In conclusion, we present a case of a brain cavernous hemangioma mimicking RIN. Physicians should be aware of the possibility for the development of brain cavernous hemangioma following SRT in patients with NSCLC.

## Disclosure

All the authors declare no conflicts of interest in association with the present report.
